# Associations of alkaline water with metabolic risks, sleep quality, muscle strength: A cross-sectional study among postmenopausal women

**DOI:** 10.1371/journal.pone.0275640

**Published:** 2022-10-31

**Authors:** Yoke Mun Chan, Zalilah Mohd Shariff, Yit Siew Chin, Sazlina Shariff Ghazali, Ping Yein Lee, Kai Sze Chan

**Affiliations:** 1 Faculty of Medicine and Health Sciences, Department of Dietetics, Universiti Putra Malaysia (UPM), Serdang, Malaysia; 2 Faculty of Medicine and Health Sciences, Research Center of Excellence Nutrition and Non-Communicable Diseases, Universiti Putra Malaysia (UPM), Serdang, Malaysia; 3 Malaysian Research Institute on Ageing, Universiti Putra Malaysia, Serdang, Malaysia; 4 Faculty of Medicine and Health Sciences, Department of Nutrition, Universiti Putra Malaysia (UPM), Serdang, Malaysia; 5 Faculty of Medicine and Health Sciences, Department of Family Medicine, Universiti Putra Malaysia (UPM), Serdang, Malaysia; 6 Faculty of Medicine, UMeHealth Unit, Universiti Malaya (UM), Kuala Lumpur, Malaysia; University of Toyama: Toyama Daigaku, JAPAN

## Abstract

Much has been claimed on the health benefits of alkaline water including metabolic syndrome (MetS) and its features with scarcity of scientific evidence. Methods: This cross-sectional comparative study was conducted to determine whether regular consumption of alkaline water confers health advantage on blood metabolites, anthropometric measures, sleep quality and muscle strength among postmenopausal women. A total of 304 community-dwelling postmenopausal women were recruited with comparable proportion of regular drinkers of alkaline water and non-drinkers. Participants were ascertained on dietary intake, lifestyle factors, anthropometric and biochemical measurements. Diagnosis of MetS was made according to Joint Interim Statement definition. A total of 47.7% of the participants met MS criteria, with a significant lower proportion of MetS among the alkaline water drinkers. The observed lower fasting plasma glucose (F(1,294) = 24.20, p = 0.025, partial η^2^ = 0.435), triglyceride/high-density lipoprotein concentration ratio (F(1,294) = 21.06, p = 0.023, partial η^2^ = 0.360), diastolic blood pressure (F(1,294) = 7.85, p = 0.046, partial η^2^ = 0.258) and waist circumference (F(1,294) = 9.261, p = 0.038, partial η^2^ = 0.263) in the alkaline water drinkers could be considered as favourable outcomes of regular consumption of alkaline water. In addition, water alkalization improved duration of sleep (F(1,294) = 32.05, p = 0.007, partial η^2^ = 0.451) and handgrip strength F(1,294) = 27.51, p = 0.011, partial η^2^ = 0.448). Low density lipoprotein cholesterol concentration (F(1,294) = 1.772, p = 0.287, partial η^2^ = 0.014), body weight (F(1,294) = 1.985, p = 0.145, partial η^2^ = 0.013) and systolic blood pressure (F(1,294) = 1.656, p = 0.301, partial η^2^ = 0.010) were comparable between the two different water drinking behaviours. In conclusion, drinking adequate of water is paramount for public health with access to good quality drinking water remains a critical issue. While consumption of alkaline water may be considered as a source of easy-to implement lifestyle to modulate metabolic features, sleep duration and muscle strength, further studies are warranted for unravelling the precise mechanism of alkaline water consumption on the improvement and prevention of MetS and its individual features, muscle strength and sleep duration as well as identification of full spectrum of individuals that could benefit from its consumption.

## Introduction

Alkaline water has higher pH than normal drinking water, contains alkaline minerals and negative oxidation reduction potential. Several methods can be used to activate water such as electrolysis, light irradiation, ultra-sonication, treatment with a magnetic field, bubbling with gases, collision, strong water flow, and treatment with specific mineral or rocks [1]. Despite human body pH is tightly regulated, the habitual consumption of mineral water or beverages with added bicarbonate has been shown to have beneficial effects in terms of increasing of urinary pH [2–4].

The effectiveness of alkaline water has gained increased recognition in health and nutrition. The application of alkaline water in the field of agriculture and medical care field was first initiated in the 1954 and 1960, and recognized for its beneficial effect on chronic diarrhea, indigestion, abnormal gastrointestinal fermentation, antacid and hyperacidity [1]. Several studies were conducted to assess the effectiveness of alkaline water in reducing the risk of metabolic syndrome (MS) or its traits [5–14] or other health outcomes [10–11, 15], with conflicting results reported.

Earlier studies found inverse association between cardiovascular diseases with increased consumption of water containing the mineral salts of calcium and magnesium [16–18], especially among the women [19]. Case-control study also demonstrated that consumption of water greater than 8mg/L of mineral salt, magnesium was associated with reduced risk of mortality from the myocardial infarction [20]. Besides, epidemiological studies in Sweden also demonstrated that consumption of water with magnesium and bicarbonate with concentration of 110mg/L were at lower risk of myocardial infarction [21], which was attributed to the decreased of urinary excretion of minerals, regulated by acid conditions in the body. Clinical study intervening mild hypertensive patients with drinking water containing 403mg/L hydrogen carbonate abled to reduce the blood pressure [22].

With its geographical location at the tropical region, water is abundantly available in Malaysia throughout the year, with both surface and ground water are used as drinking water after necessary treatment. In the Klang Valley Malaysia (Selangor, Kuala Lumpur and Putrajaya), most of the tap water supply comes from surface water sources that include rivers, lakes and reservoirs. Nevertheless, the pollution in rivers and lakes has become worsen in the recent years. The decrease in the quality of tap water because of pollution of the global environment over time has become a major social problem, whereby concern over tap water quality has led to the expansion of water filtration plants and had encouraged the marketing of filtered water, including filtered alkaline water. Earlier studies reported that 50–85% households had water filter fitted to their kitchen supply [23, 24], depends on the geographical area. These figures are believed to be higher nowadays with the reduced confidence among consumers on tap water quality as well as the increased awareness on drinking water quality among consumers [24]. Alkaline water generation has progressed and advanced in development. Besides electrolysis, alkaline minerals, nanoparticles [25] and nanofiltration membranes [26] are new technologies applied in the production of alkaline water in the water industries. To the best of knowledge, most of the previous work on alkaline water was generated using electrolysis, with little is known on the effectiveness of alkaline water generated by other technologies.

The increasing prevalence of MS is especially evident in Asia including Malaysia. Several studies in Europe and Asia have demonstrated an association between onset of menopause and higher risk of MetS, independent of aging [27–31] in postmenopausal women. Menopausal women, with declining estrogen levels, is considered particularly vulnerable with regard to impaired sleep quality [32–35] and muscle strength [36–38].

On the other hand, despite the increase usage of alkaline water in Malaysian households, with health claims on metabolic syndrome and its metabolites, studies to date provide limited information on its evidence. This was the impetus that prompted the current investigation to compare the metabolic risks, sleep quality and muscle strength between alkaline water drinkers and non-drinkers among postmenopausal women.

## Materials and methods

This was an analytical cross-sectional study conducted on community-dwelling postmenopausal women in Kuala Lumpur and Selangor, Malaysia. A total of 304 participants comprised of 148 alkaline water drinkers and 156 non-alkaline water drinkers were recruited. While non-alkaline water drinkers were recruited from various community settings including senior citizen clubs and word of mouth, alkaline water drinkers were identified and screened from the contact list provided by the alkaline water company [CUCKOO International (MAL) Pte Ltd]. The alkaline water was produced using alkaline balls and nanofiltration concept which function to retain certain mineral such as calcium and magnesium selectively from water source. Inclusion criteria included women with at least five years postmenopausal, not on hormonal replacement therapy and absence from severe diseases. While non-alkaline water drinkers were defined as participants who have not been consuming alkaline water for at least past two months, alkaline water drinkers were eligible if they consumed alkaline water on regular basis (at least 1L/day for the past two months prior to data collection). The institutional ethics board of Universiti Putra Malaysia approved this study and written informed consent were obtained from all participants prior to study commencement with anonymity and data confidentiality guaranteed.

### Metabolic risk

Measurements, including anthropometric parameters, systolic and diastolic blood pressure, fasting blood glucose and fasting lipid profile were taken. Weight and height were measured using a calibrated digital weighing scale and stadiometer, respectively. Waist circumference was measured with a circumference measurement tape. Waist was defined as the narrowest circumference between the iliac crest and the costal margin (lower rib), and hip was the widest circumference between the waist and thigh. Trained researchers conducted all measurements with routine monitoring and quality checks. Blood pressure was measured following five minutes seated rest using automatic blood pressure monitor (Omron Matsusaka Co. Ltd, Matsusaka, Japan). Blood samples for biochemical analyses were collected from participants by venipuncture following 8 hours of fasting for fasting blood sugar (FBS), triglycerides (TG), total cholesterol (TC), high density lipoprotein cholesterol (HDL-C), and low-density lipoprotein cholesterol concentration (LDL-C), using enzymatic assay kits. Presence of metabolic syndrome was ascertained as per the Joint Interim Statement (JIS) definition [39] which requires three out of five of the following risk factors: Central obesity (waist circumference of more than 80 cm), hypertension (130 mm Hg for systolic BP or 85 mm Hg for diastolic BP) or on hypertensive medication, raised FBS (5.6 mmol/L) or on diabetic medication, raised fasting TG (1.7 mmol/L) and Low HDL-C (<1.3 mmol/L). Cardiometabolic health markers assessed were the individual MetS components included in the Joint Interim Statement.

### Sleep quality

Sleep quality of participants was evaluated using the universal recognised sleep measures, the Pittsburgh Sleep Quality Index questionnaire [40]. Besides the individual’s perception of sleep quality, participants were assessed on the duration of sleep, habitual sleep efficiency, use of sleep medication, presence of sleep latency (defined as duration used to fall asleep), sleep disturbances (defined as any reason which may affect respondent’s sleep), or daytime dysfunction to allow the determination of sleep quality. Each component was weighted equally on a 3-point Likert scale, with a score of “0” indicated no difficulty, while a score of “3” indicated severe difficulty. The score from each component was summed up to yield the sleeping quality index score, which can range from 0 (no difficulty) to 21 (severe difficulty in all areas).

As a proxy measure of muscle strength, handgrip strength (HGS) of the participants was measured by using a dynamometer on the dominant hand, following standard protocol. Prior to the measurement, participants were asked if they had known upper-extremity impairments that could influence the measurement of hand grip strength. During the measurement, participants were asked to grip the hand dynamometer with the maximum force continuously for 2 to 3 seconds on a verbal statement: ‘Squeeze as hard as you can’. Two measurements were taken with a rest break of approximately 30 seconds was given between each grip. The HGS was measured in kg to one decimal point and the average of two attempts was calculated and used in further analysis. Classification of handgrip strength was according to the cut-off value proposed by Fried et al. (2001) [41], stratified by sex and Body Mass Index of participants.

Dietary intakes of respondents were assessed using a validated semi-quantitative food frequency questionnaire adapted from the Malaysian Adult Nutrition Survey 2014 [42]. The questionnaire covers 165 food items frequently consumed among Malaysian, along with their standard portion sizes. Participants indicated the typical frequency of consumption of foods and average amount (in household measures, eg cup, bowl, spoons) to allow the estimation of food intake over the past month [43]. Portion sizes were then converted to grams, based on the published household measurement. Nutrient data (protein, phosphorus, potassium, magnesium and calcium) were then analysed using Nutritionist Pro™ Diet Analysis (Version 3.2, 2007, Axxya Systems, Stafford, TX, USA) software, with Nutrient Composition of Malaysia Foods [44] and Singapore Food Composition Database [45] as the primary databases. Dietary Acid Load of the participants was calculated using potential renal acid load (PRAL) [46], an equation based on the ionic balance of the nutrients and intestinal absorption rates of protein and four main minerals (phosphorus, potassium, magnesium and calcium) as well as the sulphate production from the protein metabolism [46] as below:

            PRAL (mEq/d) = 0.49 protein (g/d) + 0.037 phosphorus (mg/d) - 0.021 potassium (mg/d) - 0.026 magnesium (mg/d) - 0.013 calcium (mg/d)

Besides dietary acid load, dietary quality index (DQI) of the participants was ascertained according to the Healthy Eating Index for Malaysia (HEI-M), which was developed and validated among Malaysian population [47]. The DQI consists of six components which assessed the compliance of participants with the food group intake based on Malaysian Dietary Guidelines (MDG) 2020. The score for each component was ranged from 0 (lack of compliance) to 10 (full compliance), and the score was calculated proportionately for the in-between responses. The overall diet quality for participant was then determined by adding the score for each component and computing a composite score with the following formula: (total score of 6 components / 6 × 10) × 100%. Based on the composite score, diet quality was classified into poor (<51%), improvement required (51% - 80%) or good (81% and above) [47].

### Statistical analyses

Data was analysed using IBM SPSS Statistics 24 software (SPSS Inc., Chicago, IL, USA). Descriptive statistics were presented as frequency and percentage for categorical variables while as mean and standard deviation for continuous variables. Independence tests were performed to determine the mean differences on age, metabolic profile (fasting plasma glucose, systolic and diastolic blood pressures, low density lipoprotein concentration, triglycerides / HDL ratio), lifestyle characteristics (sleep quality, sleep duration), dietary quality, anthropometric measures (waist circumference, weight, BMI) and muscle strength between the alkaline water drinkers and non-drinkers. Multivariate analysis of covariance (MANCOVA) was performed to determine whether alkaline water consumption augments metabolic risks (fasting plasma glucose, systolic and diastolic blood pressures, low density lipoprotein concentration, triglycerides / HDL ratio), anthropometric measures (waist circumference, body weight), sleep duration and handgrip strength, with age and physical activity as the covariates of the model. Before the exploratory data analysis were carried out, data was cleaned to delineate any possibility of wrongly entered data, missing data or outliers. Assumptions for Independence t test and Multivariate analysis of covariance (MANCOVA), namely normality, homogeneity linearity and multicollinearity (for MANCOVA) were performed. Data normalities were verified graphically (Q-Q scatter plots) and numerically (Kolmogorov–Smirnov test, skewness and kurtosis). The homogeneity of regression slots and the equality of variances were performed using Levene’s test. Linearity of correlations between dependent and independent variables were confirmed with residual plot. Multicollinearity refers to the situation that independent variables are highly correlated. In this study, multicollinearity was examined by “variance inflation factor” (VIF) values whereby VIF value that exceeds 5 or 10 indicates a problematic amount of collinearity [48]. Besides VIF, the researchers examined the correlations between the variables considering a correlation of greater than 0.37 as large [49]. Sleep quality, % body fat and BMI were correlated closely with sleep duration (r = 0.72), waist circumference (r = 0.78) and body weight (r = 0.84), and with VIFs more than 5, respectively. Considering sleep duration (sleep quality), waist circumference (% body fat), and body weight (BMI) are structural multicollinearities, sleep quality, % body fat and BMI were removed from the MANCOVA model. All assumptions for the inferential test and the covariate were met. Statistical significance was set at p<0.05.

## Results

Mean age of participants was 68 years old (Table 1). Employment rate was low with less than 15% of the participants are working. Despite the mean MET value exceeded the recommendation of the current physical guidelines and achieved at least 600 metabolic equivalent minutes (MET minutes), which is equivalent to a minimum of 150 minutes of moderate to vigorous intensity activities or 75 minutes of vigorous intensity activities PA per week, only slightly more than half of the participants met the recommendations for physical activity. This was coupled with none of the participants was either moderately active (4000–7999 MET-min/week) or highly active (≥ 8000 MET-min/week). Mean HGS was 18.1 kg, with approximately 6 in 10 of the elderly had poor grip strength. Mean duration of sleeping was approximately 5 hour 30 minutes, with more than 40% of the postmenopausal women had sleep duration of less than 5 hours and between 5–6 hours per day, respectively. This was coupled with approximately two-third of the participants were poor sleepers. Approximately one in two participants had MS. Elevated blood pressure was the most dominant component of MS (53.0%), followed by excessive waist circumference (50.3%). Approximately 60% and 40% of the participants had abnormal serum HDL and triglycerides, respectively, which out-numbered the proportion of participants with elevated blood glucose.

**Table 1:** Comparison of characteristics between alkaline water drinkers and non-drinkers (n = 304).

Variables	Alkaline water drinkers (n = 148)	Non-alkaline water drinkers (n = 156)	Overall (n = 304)	p value*
**Age (years)**	67.8 ± 6.4	68.4 ± 5.5	68.1 ± 5.9	0.074
**Years of education**	10.8 ± 3.6	10.6 ± 4.1	10.7 ± 3.9	0.085
**Employed**	23 (15.5)	18 (11.5)	41 (13.5)	0.055
**Monthly family income (MYR)**	6542±2525	5938±3206	6232±2488	0.069
**Body weight (kg)**	62.9 ± 7.2	64.6± 7.0	63.8 ± 7.1	0.046
**BMI (kg/m^2^)**	25.8 ± 3.5	26.1 ± 3.3	26.0 ± 3.4	0.060
**% Body fat**	40.6±5.8	44.2 ±4.8	42.4 ± 5.6	0.045
**Physical activity (MET-min/week)**	823±426	934±501	880 ±469	0.058
**Insufficient active (<600 MET-min/week)**	66 (44.5)	77 (49.4)	143 (47.0)	0.066
Low active (600–3999 MET-min/week)**	82 (55.5)	79 (51.6)	161 (53.0)	
**Hand grip strength (kg)**	20.8±2.8	15.5±4.1	18.1 ± 4.4	0.004
**Good hand grip**	83 (56.1)	40 (25.6)	123 (40.5)	0.002
Poor hand grip*	65 (43.9)	116 (74.4)	181 (59.5)	
**Duration of sleep (minutes)**	334±62	275± 59	304± 67	0.008
**< 5 hour per day**	48 (32.4)	89 (57.1)	137 (45.1)	0.012
**5–6 hour per day**	74 (50.0)	52 (33.3)	126 (41.4)	
**> 6 hour per day**	26 (17.6)	15 (9.6)	41 (13.5)	
**Sleep Quality Score**	3.7 ± 2.6	6.5 ± 3.1	5.14 ± 2.4	0.009
**Poor Sleeper**	81 (54.7)	114 (73.1)	195 (64.1)	< .008
**Good Sleeper**	67 (45.3)	42 (26.9)	109 (35.6)	
**Dietary Quality Score**	60.9 ± 8.23	59.6 ± 6.58	60.2 ± 4.9	0.120
**Dietary Acid Load**	20.4 ± 7.5	24.5 ± 3.3	22.5 ± 6.08	0.072
**Presence of Metabolic Syndrome**	61 (41.2)	84 (53.8)	145 (47.7)	0.041
**SBP (mmHg)**	136 ±24	138±21	137±23	0.061
**DBP (mmHg)**	92 ±11	97± 7	95 ± 10	0.075
**Elevated blood pressure**	76 (51.3)	85 (54.5)	161(53.0)	0.058
**Waist Circumference (cm)**	77.3 ±11.3	85.2±10.1	81.3±11.4	0.034
**Elevated Waist Circumference**	61 (41.2)	92 (59.0)	153 (50.3)	0.038
**LDL (mmol/L)**	3.68 ±0.48	3.82 ±0.43	3.75±0.46	0.074
**Elevated LDL**	92 (62.1)	111 (71.2)	203 (66.8)	0.157
**HDL (mmol/L)**	0.83 ± 0.18	0.82 ±0.21	0.82 ±0.20	0.062
**Low HDL**	110 (74.3)	63 (40.4)	173 (56.9)	0.068
**TG (mmol/L)**	1.60 ±0.39	1.89 ±0.34	1.75±0.40	0.029
**Elevated serum TG**	51 (34.9)	64 (41.0)	115 (37.8)	0.065
**FBG (mmol/L)**	4.69 ±1.03	5.11 ±1.02	4.90 ±1.04	0.038
**Elevated FBG**	38 (25.7)	50 (32.1)	88 (28.9)	0.056
**TG/HDL**	2.03 ± 0.65	2.51 ± 0.14	2.27 ± 0.2f5	0.035

Data were presented as mean ± standard deviation or n (%)

* AWGS ** Classified according to Kyu et al. (2016) [50] *** comparison between two groups were made either using Independence t test or chi-square independence test.

Overall, there were no significant differences on the mean age, years of education, employment status and gross monthly family income between the regular alkaline water drinkers and non-drinkers. Regular alkaline water drinkers had significant lower body weight and % body fat. On the other hand, they have significant higher muscle strength than their non-alkaline water drinker counterparts. Meanwhile, non-alkaline water drinkers had poorer sleep quality and significant shorter duration of sleep, with regular alkaline water drinkers had significant longer sleep duration of approximately 70 minutes per day. There was no significant difference on physical activity between the two groups (t = 1.87, *p*>0.05, df = 304). Dietary quality scores and dietary acid load were comparable between the non-alkaline water drinkers and their counterparts. With regards to metabolic syndrome, there was smaller proportion of regular alkaline water drinkers presented with metabolic syndrome (41.2% vs 53.8%). While diastolic blood pressure was comparable between the two groups, non-alkaline water drinkers had significant higher waist circumference and fasting blood glucose (*p*<0.05). It is noteworthy that TG/HDL ratio was significantly lower among alkaline water drinkers (t = 2.01, *p*<0.05, df = 304), despite a comparable of serum HDL between the groups, attributed to the higher serum triglycerides among the non-alkaline water drinkers (t = 2.30, *p*<0.05, df = 304).

A one-way multivariate analysis of covariance (MANCOVA) was performed to determine the influence of drinking behaviour (alkaline water drinkers vs non-drinkers) on the nine primary outcome variables: i) fasting plasma glucose, ii) TG/HDL, iii) LDL, iv) SDP, v) DBP, vi) waist circumference, vii) body weight, viii) sleep duration, and ix) hand grip strength, after controlling for age and physical activity level as covariates in the model (Table 2).

**Table 2:** Influence of drinking behaviour (alkaline water drinkers vs non-drinkers) on metabolite risks (FPG, TG/HDL, LDL, SBP, DBP), anthropometric parameters (body weight, WC), sleep duration and handgrip strength.

	FPG		TG/HDL		LDL		SBP		DBP		WC		Body weight		Sleep Duration		Handgrip strength	
	F	p	F	p	F	p	F	p	F	p	F	p	F	p	F	p	F	p
Intercept	2.54	0.047	0.23	0.65	0.21	0.66	0.08	0.76	0.42	0.45	0.54	0.40	0.13	0.74	1.12	0.14	0.65	0.38
Drinking behaviour	24.20	**0.025** *	21.06	**0.023** *	1.772	0.287	1.656	0.301	7.85	**0.046** *	9.261	**0.038** *	1.985	0.145	32.05	**0.007** *	27.51	**0.011** *

* significant at p<0.05.

Age and physical activity were the covariates of the models.

Overall, the model was statistically significant, indicating differences between the two water drinking behaviour after controlling for covariates (*F*(8,296) = 3.25, *p* = 0.025, *partial η*^*2*^ = 0.310). A significant main effect of drinking behaviour (drinker vs non-drinkers) was found for FBG (*F*(1,294) = 24.20, *p* = 0.025, *partial η*^*2*^ = 0.435), TG/HDL (*F*(1,294) = 21.06, *p* = 0.023, *partial η*^*2*^ = 0.360), DBP (*F*(1,294) = 7.85, *p* = 0.046, *partial η*^*2*^ = 0.258), with no significant main effect of alkaline water drinking behaviour on LDL (*F*(1,294) = 1.772, *p* = 0.287, *partial η*^*2*^ = 0.014) or SBP (*F*(1,294) = 1.656, *p* = 0.301, *partial η*^*2*^ = 0.010). Postmenopausal women who were alkaline water drinkers had lower waist circumference (*F*(1,294) = 9.261, *p* = 0.038, *partial η*^*2*^ = 0.263), but there was no statistically significant difference between the alkaline water drinking groups on the body weight after controlling for covariates (*F*(1,294) = 1.985, *p* = 0.145, *partial η*^*2*^ = 0.013). On the other hand, there were significant main effects of drinking behaviour on sleep duration (*F*(1,294) = 32.05, *p* = 0.007, *partial η*^*2*^ = 0.451) and hand grip strength (*F*(1,294) = 27.51, *p* = 0.011, *partial η*^*2*^ = 0.448).

## Discussion

The main result of this study is that alkaline water drinking among postmenopausal women had significantly lower metabolite risks (fasting plasma glucose, TG/HDL, diastolic blood pressure, waist circumference), longer sleep duration and stronger handgrip strength. There was no significant difference on LDL, systolic blood pressure and body weight with alkaline water drinking.

This is the first study comparing features between alkaline drinkers and non-drinkers. In lieu of lacking similar study for comparison, we compared our findings on metabolic risks with prospective and interventional trials. Anti-obesity effects of alkaline water have been reported in animal models [51, 52] with inconsistency in other [53]. There is little compelling evidence on alkaline water consumption and obesity in human, with findings had been reported as both positive [5] or neutral [13]. Current findings on the obesity indexes (body weight, body weight status, % body fat and waist circumference) deserve further elaboration. While the universal proxy measure of obesity, mean BMI was comparable between the two groups, alkaline water drinkers in general has significant lower body weight, waist circumference and % body fat, however alkaline water drinking only had significant main effect on waist circumference but not other obesity indices including body weight. Body mass index and body weight do not take into account the distribution of fat mass and cannot discriminate fat mass from lean mass, which is of particular importance in older individuals, as the distribution of body fat changes with age [54], even in the absence of changes in body weight. On the other hand, waist circumference is strongly correlate with abdominal obesity and is a commonly used clinical measure of body fat distribution [55]. It was hypothesized alkaline water might have influenced the production of leptin or adiponectin, induced lipolysis in adipocytes, downregulated the expression of transcription factors in the adipogenesis pathway, or reduced lipid accumulation by affecting the expression of genes, such as fatty acid synthase and lipoprotein lipase during preadipocytes differentiation [5]. In light of the expanding global burden of obesity on socioeconomic and health care, more work is warranted to delineate the relationship between consumption of alkaline water and risk of abdominal obesity.

In the present study, we found that alkaline drinkers have lower serum fasting blood glucose and triglycerides which add evidence to the scarcity of data on this aspect in human trials. Previous human studies showed alkaline water supplementation ameliorated blood glucose [9, 12, 14] and HbA1c [14] significantly, which was incongruent with human [5, 10–11] or animal studies [53, 56–58]. Earlier, it was hypothesized that alkaline water could substantially increase the activity of hexokinase, which is a pivotal enzyme inducing the reduction of blood glucose levels [59]. More recently, evidence is growing that oxidative stress plays a key role in the aetiology and pathophysiology of diabetes [60–62], involved in chronic hyperglycaemia-induced insulin resistance [63] and vascular complications [64]. The actual protective mechanism of alkaline water is yet to be elucidated but it could be attributed to its active atomic hydrogen that has a high reducing ability which may participate in redox reactions and contributing to increasing levels of antioxidants [65]. This was confirmed by recent study that intervention of alkaline water on patients with diabetes mellitus was associated with lower level of oxidative stress and inflammatory markers [9], which represents the first human trial on alkaline water supplementation, with more evidence available from animal models [51, 66] or at laboratory testing [67]. Different from Gadek et al. (2006) [14], Rias et al. (2019) [9] and Siswantoro, Purwanto & Sutomo (2017) [12] whose participants were patients with diabetes mellitus, our participants were entirely healthy postmenopausal women, hence this finding shed light on the possible health benefit of alkaline water on healthy individual, which should be confirmed with more human trials. It is worth noting that earlier mentioned clinical trials were relatively short (ranged from six days to eight weeks), at which the positive outcomes should be confirmed with longer intervention or prospective studies.

Studies on the effectiveness of alkaline water on lipid profiles had been scarce. Our findings did not find significant differences on HDL profiles between alkaline water drinkers and non-drinkers. These findings echo recent studies in Korea and Indonesia [5, 6]. Although mean LDL concentration was comparable between alkaline and non-alkaline water drinkers at bivariate analysis, alkaline water drinking favours lower LDL at the MANCOVA analysis after controlling for covariates. On the other hand, the role of alkaline water had been rather consistently positive for triglycerides in animal models [68–70], which was absent in human studies. Our finding on serum triglyceride is consistent with some previous accounts [7, 56, 71] and discrepant with others [5, 6, 10, 72]. Cardiovascular disease is the leading cause of death in women at advanced age, who are affected a decade later compared to men [73], possibly attributed to the deterioration of lipid profile which becomes more atherogenic among postmenopausal women than their premenopausal counterparts [74]. Numerous studies showed elevated triglyceride increased risk of coronary artery disease in postmenopausal women [75] and play a key role in predicting cardiovascular disease in women [76]. On the other hand, growing body of evidence is suggesting the ratio of TG/HDL-C as an easily obtainable atherogenic marker [77] and predictor of all‐cause mortality [78]. Elevated ratio of TG/HDL has also been associated with poor cardiovascular outcomes in patients with chronic kidney, silent brain infarct, ischemic stroke, cardiovascular disease [79–82] and mortality [83]. Our findings were the only study indicating consumption of alkaline water led to lower ratio of TG/HDL, which should be confirmed with further prospective studies. It is imperative to highlight that absolute or ‘global’ approach to assessing and managing CVD risk has the potential to prevent twice as many deaths from coronary heart disease when compared with treating individual risk factors, such as blood pressure or cholesterol. More works are warranted to delineate the effectiveness of alkaline water on lipid profiles as well as CVD risks.

Alkaline water drinking has mixed findings on blood pressures, whereby significant main effect was only reported for diastolic but not systolic blood pressure. Inconsistency in findings were documented in earlier studies [13, 84–86]. The later researchers speculated the use of alkaline water as the hemodialysis solution may counteract with the action by radical oxygen species and potent vasoconstrictor such as peroxynitrite and lead to vasodilation [84]. A recent animal study reported alkaline water may downregulating oxidative stress and inhibiting inflammation, leading to lower blood pressure [87]. Essential elements such as calcium and magnesium are generally higher in filtered alkaline water, which is speculated to contribute to lower blood pressure as well. As hypertensive is one of the major causes of morbidity and mortality and affects a considerable proportion of the population, with many more are underdiagnosed, the use of alkaline water can be considered as a simple lifestyle modification to modulate blood pressure. Before such recommendation is made, extensive and quality studies are needed.

Our findings that drinkers of alkaline water had significant longer sleep duration deserve more in-depth elaboration, in lieu of the societal trend toward less sleep and poorer quality sleep is a common feature in many developed countries [88–90] and developing countries [91, 92]. Poor sleep quality including sleep disturbance and short sleep duration are often associated with unfavourable health outcomes including mental, physical and cognitive health [93]. Recent study showed that alkaline water consumption improved sleep quality of adults in Japan [10], however comparison between group was not available, and make it difficult to determine if any effect is due to the different intervention received or simply a result of practice. Earlier studies showed elevation of inflammation and oxidative stress are common features among sleep disordered populations [94] while consumption of kiwi (rich in vitamin A, vitamin E and serotonin) [95] and tart cherry juice (rich in vitamins A and C) [96] promoted better sleep, with inconsistencies exist [97]. More recent studies reported a direct relationship between sleep duration and quality, with fruit and vegetable intake [98, 99] or polyphenol-rich foods (i.e., black tea and cocoa products) [100, 101], which are known antioxidants. Acknowledging the size of the sleep problem in the modern societies and the scarcity of data, more works are needed to delineate the positive effect of alkaline water on sleep quality.

In the present study, with the comparable dietary quality scores and dietary acid load between the two groups, it is reasonable to assume that any beneficial effects of alkalinity towards metabolic are likely to attribute by the alkaline water consumption. One of the study limitations was we did not analyse or incorporate the minerals content of water drank in the calculation of dietary acid load of participants as the mineral contents of tap water varies according to geographical locations and depends on the mineral compositions of the soil and pollutants such as heavy metal [102]. Future studies should also consider the level of antioxidants present in alkaline water. We acknowledge that the alkaline water in previous published work was produced by electrolysis. The investigated alkaline water was acquired using a different mechanism, namely alkaline balls and nanofiltration membrane concept which function to retain certain mineral such as calcium and magnesium selectively from water source, reduces [H+] and increases [OH-], and leads to an overall rising of water pH. These preliminary data suggested consumption of alkaline water produced using other technology concept demonstrated comparable results with electrolyzed reduced alkaline water. Considering the limited information on its evidence, future work is warranted to compare the effectiveness of different water treatment in acquiring alkaline water. The cross-sectional study design limits our ability to draw predictive conclusions, hence longer intervention or prospective studies are needed to delineate the benefits of alkaline water drinking in the future studies.

## Conclusions

Alkaline water consumption may be considered as a source of easy-to implement lifestyle to modulate metabolic features. However further studies are warranted for unravelling the full spectrum of individuals that could benefit from its consumption. Additionally, the precise mechanism of alkaline water consumption on the improvement and prevention of diseases such as metabolic syndrome and its individual features are not fully elucidated, hence the necessity of studies addressing its broad effect on health status improvement and mechanism merit further studies.

## Supporting information

S1 Questionnaire (XLSX)
